# Protective Effects of Hong Shan Capsule against Lethal Total-Body Irradiation-Induced Damage in Wistar Rats

**DOI:** 10.3390/ijms160818938

**Published:** 2015-08-12

**Authors:** Jianzhong Li, Jing Xu, Weiheng Xu, Yang Qi, Yiming Lu, Lei Qiu, Zhenlin Hu, Zhiyong Chu, Yifeng Chai, Junping Zhang

**Affiliations:** 1 School of Pharmacy, Second Military Medical University, Shanghai 200433, China; E-Mails: xujing0601103@live.cn (J.X.); xuweiheng7114@163.com (W.X.); qyangangle@sina.com (Y.Q.); bluesluyi@sina.com (Y.L.); qlcong021@163.com (L.Q.); zhenlinhu@hotmail.com (Z.H.); yfchai@smmu.edu.cn (Y.C.); 2 Department of Pharmacy, East Hospital, Dongji University, Shanghai 200085, China; 3 Department of Preventive Medicine, Naval Medical Research Institute, Shanghai 200433, China; E-Mail: zhiyongchu@tom.com

**Keywords:** Hong Shan capsule (HSC), lethal total-body irradiation, radioprotection, gene expression, signaling pathways

## Abstract

Hong Shan Capsule (HSC), a crude drug of 11 medicinal herbs, was used in clinical practice for the treatment of radiation injuries in China. In this study, we investigated its protection in rats against acute lethal total-body irradiation (TBI). Pre-administration of HSC reduced the radiation sickness characteristics, while increasing the 30-day survival of the irradiated rats. Administration of HSC also reduced the radiation sickness characteristics and increased the 30-day survival of mice after exposure to lethal TBI. Ultrastructural observation illustrated that the pretreatment of rats with HSC significantly attenuated the TBI-induced morphological changes in the different organs of irradiated rats. Gene expression profiles revealed the dramatic effect of HSC on alterations of gene expression caused by lethal TBI. Pretreatment with HSC prevented differential expression of 66% (1398 genes) of 2126 genes differentially expressed in response to TBI. Pathway enrichment analysis indicated that these genes were mainly involved in a total of 32 pathways, such as pathways in cancer and the mitogen-activated protein kinase (MAPK) signaling pathway. Our analysis indicated that the pretreatment of rats with HSC modulated these pathways induced by lethal TBI, such as multiple MAPK pathways, suggesting that pretreatment with HSC might provide protective effects on lethal TBI mainly or partially through the modulation of these pathways. Our data suggest that HSC has the potential to be used as an effective therapeutic or radio-protective agent to minimize irradiation damage.

## 1. Introduction

Radiation toxicity is a kind of physical stress that humans risk in events such as nuclear pollution, radiation therapy for cancer, and space flights [1,2,3]. Radiation exposure increases oxidative pressure and induces further damage such as DNA lesions, cell death, cancer, and other diseases. Radio-protective agents are administered either before or after radiation exposure to minimize radiation toxicity. Several compounds have been reported to offer the potential for radiation protection, but most of them are not suitable for clinical application due to toxicity and poor specificity [4,5,6]. This warrants the development of suitable radio-protective agents with minimum toxicity that can be used under occupational as well as clinical conditions.

A number of medicinal plants and their extracts evaluated for the radio-protective efficacy have shown protective effects against radiation damage [7]. In traditional Chinese medicine, it is a common practice to use many components for treating diseases. Hong Shan Capsule (HSC) has been used in clinical practice for the treatment of radiation injuries in China. HSC is composed of 11 medicinal herbs, including *Cornus officinalis*, *Fructus crataegi*, *Millettia dielsiana*, *Pericarpium Citri reticulatae*, *Radix Ginseng rubra*, *Rehmannia glutinosa*, *Radix astragali*, *Radix Paeoniae lactiflorae*, *Semen coicis*, *Rhizoma anemarrhenae*, and *Rhizoma Ligustici chuanxiong*. Though several ingredients and HSC were reported to have the radiation protective effect [8,9,10,11,12,13,14,15], clarifying the mechanism of the pharmacological action of HSC remains a great challenge due to the haziness of the active compounds and the unknown synergistic actions of multiple components. In this study, we mainly investigated its protective effects against lethal TBI in Wistar rats.

## 2. Results

### 2.1. Effect of HSC on 30-Day Survival

**Figure 1:**
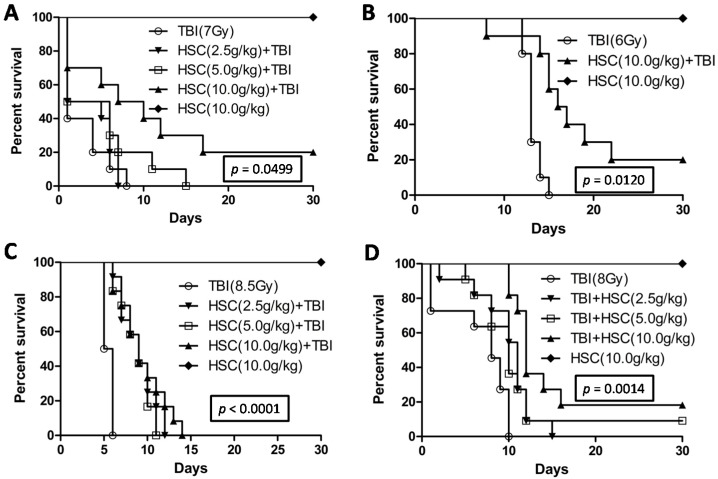
Comparison of survival curves by using the Kaplan–Meier method. (**A**) 30-day survival of rats (*n* = 10 per group) pretreated with HSC (2.5, 5, and 10 g/kg) before exposure to 7 Gy TBI. The survival curves were significantly different as predicted by the log rank test. (*p* = 0.0499); (**B**) 30-day survival of rats (*n* = 10 per group) pretreated with HSC (10 g/kg) before exposure to 6 Gy TBI. The survival curves were significantly different as predicted by the log rank test. (*p* = 0.0120); (**C**) 30-day survival of mice (*n* = 12 per group) pretreated with HSC (2.5, 5 and 10 g/kg) before exposure to 8.5 Gy TBI. The survival curves were significantly different as predicted by the log rank test. (*p* < 0.0001); and (**D**) 30-day survival of mice (*n* = 11 per group) treated with HSC (2.5, 5 and 10 g/kg) after exposure to 8 Gy TBI. The survival curves were significantly different as predicted by the log rank test. (*p* = 0.0014).

The radiation control group exhibited signs of radiation sickness such as reduced intake of food and water, weight loss, lethargy, diarrhea, and epilation, with a median survival of only one day and complete mortality within eight days (Figure 1A). HSC (10 g/kg) alone did not induce any noticeable signs of toxicity within 30 days. On the other hand, rats that were administered different graded concentrations of HSC for three consecutive days prior to the exposure of acute lethal TBI (7 Gy) had reduced signs of radiation sickness and improved survival rates. Different concentrations of HSC were found to offer the same protection to rats against radiation-induced toxic effects by increasing the median survival period to 3, 3.5, and 8.5 days, respectively (Figure 1A). Pre-administration of 10 g/kg of HSC reduced the radiation sickness characteristics while increasing the 30-day survival of the irradiated rats by 20% (Figure 1A). The log rank test indicated that the survival curves were significantly different (*p* = 0.0499). In addition, when rats were subjected to exposure to a lethal dose of 6 Gy radiation, 10 g/kg of HSC also increased 30-day survival by 20% (Figure 1B). The survival curves were significantly different as predicted by the log rank test (*p* = 0.0120). The survival curves (Figure 1C) indicated that the pre-administration of HSC was also found to offer the same protection to Institute of Cancer Research (ICR) mice against acute lethal TBI (8.5 Gy)-induced toxic effects by increasing the median survival period. The log rank test indicated that the survival curves were significantly different (*p* < 0.0001).

Encouraged by the above results, the radio-protective ability of HSC was also evaluated by administering it 30 min after exposure to a lethal dose (8 Gy) of TBI. Mice that were administered with different concentrations of HSC for three consecutive days after TBI had reduced signs of radiation sickness and increased median survival (Figure 1D). The administration of 5 and 10 g/kg of HSC increased the 30-day survival of the irradiated mice by 9% and 18%, respectively, confirming its radio-protective ability (Figure 1D). The log rank test also indicated that the survival curves were significantly different (*p* = 0.0014).

### 2.2. Effect of HSC on Radiation-Induced Tissue Injury Using Transmission Electron Microscopy

**Figure 2:**
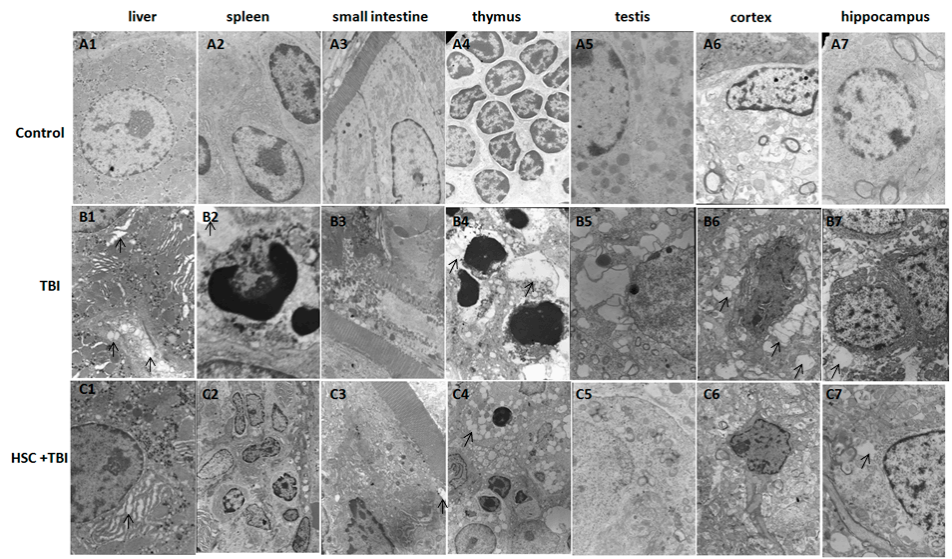
Effect of HSC on radiation-induced tissues injury at one day after exposure to lethal TBI (6.5 Gy) using transmission electron microscopy. (**A1**–**7**) Representative photographs of non-irradiated rats; (**B1**–**7**) Representative photographs of 10 TBI rats. Cell swelling and large amounts of cytoplasmic vacuoles (arrows) were observed in the different tissues at one day after exposure to lethal TBI (6.5 Gy); (**C1**–**7**) Representative photographs of 10 irradiated rats pretreated with HSC (10 g/kg) once each day for three consecutive days before exposure to 6.5 Gy TBI. HSC pretreatment attenuated the radiation-induced tissue injury such as cytoplasmic vacuoles (arrows). (Uranyl acetate and lead citrate staining, 3000×).

To observe the possible protective or therapeutic effect of HSC on radiation-induced tissue injury in rats, transmission electron microscopy was performed. Rats received 10 g/kg/day of HSC or water for three days prior to radiation. Significant pathological changes occurred at one day after acute lethal TBI (6.5 Gy). Damage in the liver, spleen, small intestine, thymus, testis, cortex, and hippocampus such as cytoplasmic vacuolization, dilatation of the endoplasmic reticulum, and destruction of mitochondria, as well as damage to the cellular membrane, were observed (Figure 2B1–7). In addition, the morphological signs of apoptosis were frequently detected in the splenocytes. Apoptotic splenocytes showed the marginal condensation of chromatin onto the nuclear lamina (Figure 2B2). Vacuoles and mitochondrial swelling were decreased by HSC in the different organs (Figure 2C1–7). Cell apoptosis was also significantly attenuated by HSC in the spleen (Figure 2C2). These results illustrated that pretreatment of rats with HSC before exposure to acute lethal TBI significantly attenuated the radiation-induced morphological changes in the irradiated different organs, confirming the protective or therapeutic effect of HSC on radiation-induced tissue injury.

### 2.3. HSC Prevents Radiation-Induced Differential Expression of Many Genes

**Figure 3:**
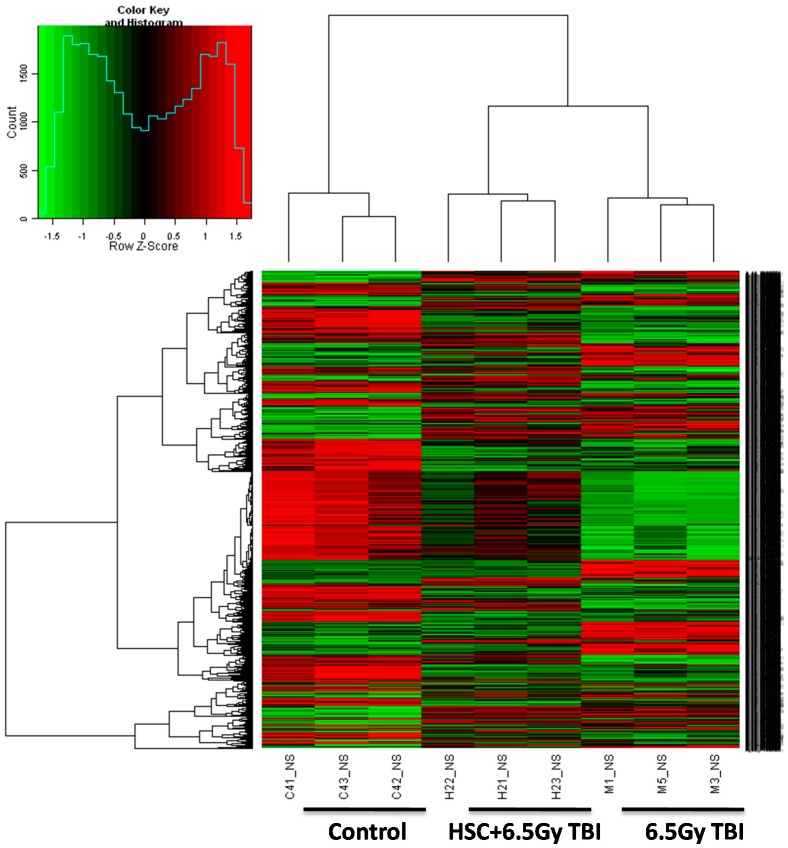
Cluster analysis of individual rats according to the profile of gene expression examined. Genes are organized by hierarchical clustering based on overall similarity in expression patterns. Red represents relative expression greater than the median expression level across all samples, and green represents an expression level lower than the median. Black indicates intermediate expression. Rats from the same treatment group (6.5 Gy TBI, 10 g/kg HSC + 6.5 Gy TBI) were the closest.

The liver was considered to be a radiosensitive organ [16]. Vacuoles and mitochondrial swelling were significantly decreased by HSC in the liver (Figure 2C1), indicating that HSC ameliorated TBI-induced liver tissue injury. To further investigate the potential molecular basis of the protective effects of HSC on irradiation damage, gene expression analysis was conducted on rats’ liver tissue using microarrays. A heatmap was generated (Figure 3), representing 3428 transcripts (probesets) comparing significantly differentially regulated genes (*p* < 0.01) for each treatment group (Radiation, HSC + Radiation) *versus* untreated controls by using one-way analysis of variance (one-way ANOVA) (additional file 1). Cluster analysis of the microarray results revealed that three rats from the same group were the closest, indicating that all the experimental data in this study were reliable.

A combined algorithm with simple *t*-test and fold change was used to find differentially expressed genes. By a threshold of *p* < 0.05 and absolute fold change (FC) ≥ 1.5, 2126 differentially expressed genes (transcripts) were selected by comparison between radiation and wild-type (control) samples (additional file 2). Among the genes originally displaying FC ≥ 1.5, 1067 were down-regulated and 1059 up-regulated. In the pretreatment with HSC groups, only 728 out of 2126 genes were still differentially expressed in response to radiation. These results indicated that the treatment with HSC prevented the radiation-induced differential expression of 66% (1398 genes) of genes (additional file 3). We focused on these genes for which radiation-induced alteration of expression was abolished or attenuated by HSC pretreatment, as these genes might be regulated by HSC and involved in the protective effects against radiation.

**Figure 4:**
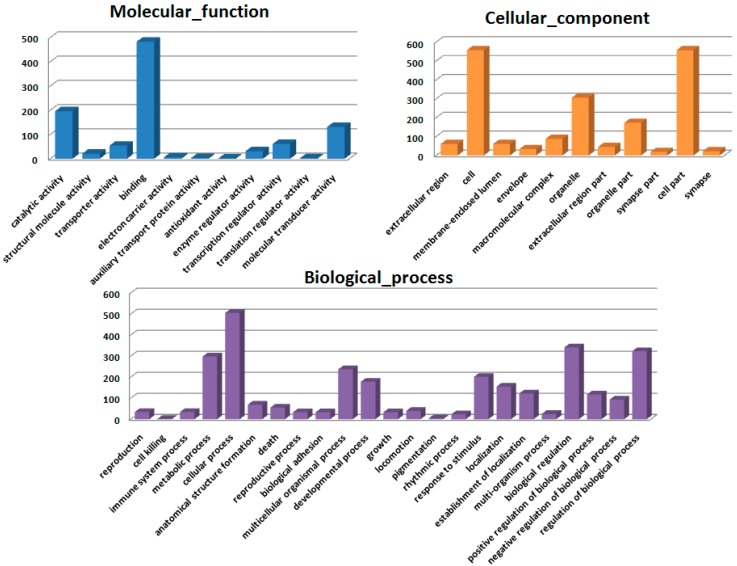
GO ontology analysis of genes for which radiation-induced alteration of expression was abolished or attenuated by HSC. Gene ontology (level 2) for the transcriptome under Molecular_function, Cellular_component, and Biological_process.

Gene ontology (GO) annotation was further performed for these genes in terms of molecular function, cellular components, and biological processes. Distribution of the genes in different GO categories at level 2 was shown in Figure 4. The highly represented GO terms were binding and catalytic activity for molecular function, cell part, and cell for cellular component, cellular process, and biological regulation for biological process.

Pathway enrichment analysis indicated that these genes were mainly involved in a total of 32 pathways, such as olfactory transduction, neuroactive ligand-receptor interaction, pathways in cancer, the calcium signaling pathway, vascular smooth muscle contraction, cytokine-cytokine receptor interaction, the mitogen-activated protein kinase (MAPK) signaling pathway, the peroxisomal proliferator-activated receptor (PPAR) signaling pathway, the gonadotropin-releasing hormone (GnRH) signaling pathway, the Wnt signaling pathway, the janus kinase-signal transducers and activators of the transcription (Jak-STAT) signaling pathway, the Notch signaling pathway, purine metabolism, glutathione metabolism, and fatty acid metabolism (Table 1). For example, the 10 genes presented in Table 2 were involved in the MAPK pathway. Eight of the gene expressions were evidently up-regulated by radiation and these up-regulations were abolished or attenuated by pretreatment with HSC. Nine genes were involved in the PPAR signaling pathway (Table 3). Seven of the gene expressions were evidently up-regulated by radiation and these up-regulations were abolished or attenuated by pretreatment with HSC. Eight genes were involved in the GnRH signaling pathway (Table 4). Seven of the gene expressions were evidently up-regulated by radiation and these up-regulations were abolished or attenuated by pretreatment with HSC. Six genes were involved in the Wnt signaling pathway (Table 5). All of these gene expressions were evidently up-regulated by radiation and these up-regulations were abolished or attenuated by pretreatment with HSC. Five genes were involved in the Notch signaling pathway (Table 6). Four of the gene expressions were evidently up-regulated by radiation and these up-regulations were abolished or were attenuated by pretreatment with HSC.

**Table 1:** KEGG pathway analysis of genes for which radiation-induced alteration of expression was abolished or attenuated by HSC.

Name	Hits	Percent	Enrichment Test *p* Value
Olfactory transduction	41	3.96%	0.0064
Neuroactive ligand-receptor interaction	16	5.63%	0.0043
Pathways in cancer	15	4.66%	0.0251
Calcium signaling pathway	13	6.99%	0.0017
Vascular smooth muscle contraction	11	9.09%	5.00 × 10^−4^
Gap junction	10	11.24%	2.00 × 10^−4^
Cytokine-cytokine receptor interaction	10	4.83%	0.0494
MAPK signaling pathway	10	3.72%	0.1465
Systemic lupus erythematosus	9	6.77%	0.0101
Purine metabolism	9	5.39%	0.035
PPAR signaling pathway	9	11.39%	4.00 × 10^−4^
Tight junction	8	6.02%	0.0269
GnRH signaling pathway	8	8.08%	0.0058
Axon guidance	8	6.30%	0.0214
ECM-receptor interaction	7	9.21%	0.005
Protein digestion and absorption	6	7.69%	0.0195
Wnt signaling pathway	6	3.90%	0.2176
Hematopoietic cell lineage	6	7.59%	0.0205
Adipocytokine signaling pathway	6	8.57%	0.0124
Jak-STAT signaling pathway	6	4.08%	0.1902
Acute myeloid leukemia	6	10.71%	0.0047
Notch signaling pathway	5	10.00%	0.0124
Glutathione metabolism	5	8.93%	0.0187
Fatty acid metabolism	5	10.42%	0.0106
Bile secretion	5	6.76%	0.0494
Starch and sucrose metabolism	4	8.51%	0.0394
Staphylococcus aureus infection	4	8.16%	0.0444
Mineral absorption	4	8.70%	0.037
Galactose metabolism	4	16.00%	0.0058
Circadian rhythm	4	19.05%	0.0033
Carbohydrate digestion and absorption	4	10.53%	0.021
ABC transporters	4	8.89%	0.0347

**Table 2:** The 10 genes that were involved in the MAPK pathways.

Gene ID	Symbol	Description	Fold Change	
			TBI	HSC + TBI
24577	Myc	Myelocytomatosis oncogene	3.41	2.03
29692	Pla2g2a/sPLA2	Phospholipase A2, group IIA	5.65	4.25
501563	Prkx	Protein kinase, X-linked	1.58	1.32
54234	Cacna1e	Calcium channel, voltage-dependent, R type, α 1E subunit	2.14	1.10
682930	Cacna1s	Calcium channel, voltage-dependent, L type, α 1S subunit	3.30	1.04
301343	Map4k4	Mitogen-activated protein kinase kinase kinase kinase 4	1.75	1.37
64666	Taok2	TAO kinase 2	1.66	0.93
79255	Atf4	Activating transcription factor 4	1.54	1.11
365057	Ask1	Similar to apoptosis signal-regulating kinase 1	0.42	0.85
59109	Ntrk1	Neurotrophic tyrosine kinase, receptor, type 1	0.29	0.58

**Table 3:** The nine genes that were involved in the PPAR signaling pathway.

Gene ID	Symbol	Description	Fold Change	
			TBI	HSC + TBI
25682	Ppard	Peroxisome proliferator-activated receptor delta	1.88	0.89
94340	Acsl5	Acyl-CoA synthetase long-chain family member 5	1.67	1.34
266674	Cyp4a8	Cytochrome P450, family 4, subfamily a, polypeptide 8	3.35	2.56
501072	Acaa1b	Acetyl-Coenzyme A acyltransferase 1B	1.85	1.35
50549	Cyp4a1	Cytochrome P450, family 4, subfamily a, polypeptide 1	1.93	1.36
25756	Cpt1b	Carnitine palmitoyltransferase 1b, muscle	2.11	1.39
79223	Gk	Glycerol kinase	1.52	1.32
25747	Ppara	Peroxisome proliferator activated receptor alpha	0.66	0.99
24552	Me1	Malic enzyme 1, NADP(+)-dependent, cytosolic	0.67	0.74

**Table 4:** The eight genes that were involved in the GnRH signaling pathway.

Gene ID	Symbol	Description	Fold Change	
			TBI	HSC + TBI
25433	Hbegf	Heparin-binding EGF-like growth factor	2.50	1.46
81686	Mmp2	Matrix metallopeptidase 2	1.74	1.50
682930	Cacna1s	Calcium channel, voltage-dependent, L type, α 1S subunit	3.30	1.04
29692	Pla2g2a	Phospholipase A2, group IIA	5.65	4.25
83805	Src	V-src avian sarcoma (Schmidt-Ruppin A-2) viral oncogene homolog	1.62	1.16
79255	Atf4	Activating transcription factor 4	1.54	1.11
501563	Prkx	Protein kinase, X-linked	1.58	1.32
25679	Itpr3	Inositol 1,4,5-trisphosphate receptor, type 3	0.58	0.84

**Table 5:** The six genes that were involved in the Wnt signaling pathway.

Gene ID	Symbol	Description	Fold Change	
			TBI	HSC + TBI
114850	Wnt7a	Wingless-type MMTV integration site family, member 7A	1.63	0.99
64512	Fzd2	Frizzled family receptor 2	2.95	2.16
293649	Lrp5	Low density lipoprotein receptor-related protein 5	1.57	1.12
25682	Ppard	Peroxisome proliferator-activated receptor Δ	1.88	0.89
501563	Prkx/PKA	Protein kinase, X-linked	1.58	1.32
24577	Myc	Myelocytomatosis oncogene	3.41	2.03

**Table 6:** The five genes that were involved in the Notch signaling pathway.

Gene ID	Symbol	Description	Fold Change	
			TBI	HSC + TBI
79225	Hes5	hes family bHLH transcription factor 5	2.28	1.70
360801	Ncor2	nuclear receptor co-repressor 2	1.61	1.31
116462	Ptcra	pre T-cell antigen receptor α	1.81	1.08
362268	Rbpjl	recombination signal binding protein for immunoglobulin kappa J region-like	2.29	1.08
300802	Aph1b	APH1B gamma secretase subunit	0.56	0.70

**Figure 5:**
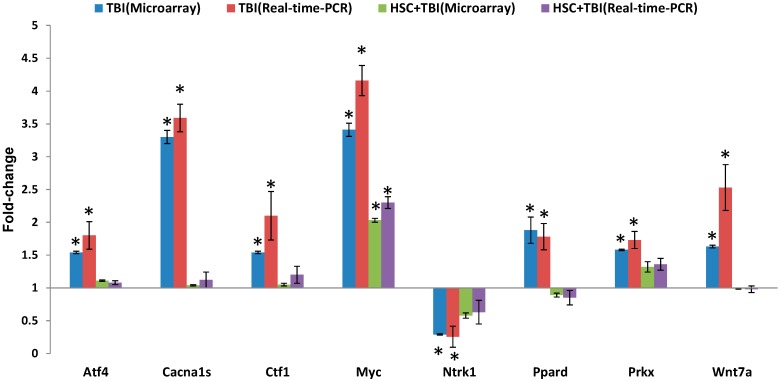
Quantitative real-time PCR confirmation of the microarray data. qRT-PCR was performed on eight genes where TBI-induced alteration of expression was abolished or attenuated by 10 mg/kg HSC pretreatment. RNA samples of different groups (*n* = 5 per group) were prepared 24 h after exposure to 6.5 Gy TBI. Gene expression levels are shown as the mean normalized to the expression of the housekeeping gene beta-actin. Each sample was measured in triplicate. Columns, the mean of three or four rats in the microarray experiment or the mean of five rats in PCR; bars, SD. * indicates statistical significance compared to control (non-irradiation) by *t*-test, * *p* < 0.05. Comparison of fold change produced by microarray with relative expression ratio obtained from real-time PCR with good concordance.

To validate the consistency of microarray analysis in the present study, we compared gene expression levels of eight selected genes (Myc (MAPK pathways and Wnt signaling pathway), Ntrk1 (MAPK pathways), Atf4 (MAPK pathways and GnRH signaling pathway), Cacna1s (GnRH signaling pathway), Ctf1 (cardiotrophin 1, Cytokine-cytokine receptor interaction and Jak-STAT signaling pathway), Ppard (Wnt signaling pathway and PPAR signaling pathway), Wnt7a and Prkx (Wnt signaling pathway)) between microarray and real-time PCR. We determined the mean value of expression of the selected genes in five independent rats from each treatment group. This was compared with those in pooled RNA from five non-irradiated rats. The qualitative changes in gene expression levels were consistent between microarray and real-time PCR (Figure 5).

## 3. Discussion

HSC is composed of 11 crude drugs and several of them have been reported to have protective effects against radiation. For example, ginseng appeared to be a promising radio-protector for therapeutic or preventive protocols capable of attenuating the deleterious effects of radiation on normal human tissue, especially for cancer patients undergoing radiotherapy [14]. Catalpol (a main bioactive component in the roots of *Rehmannia glutinosa*) decreased plasma malondialdehyde (MDA) intestinal 8-hydroxydeoxyguanosine (8-OHdG) levels and increased plasma endogenous antioxidants and peripheral white blood cells and platelets *in vivo*, which suggested that catalpol possessed notable radio-protective activity by reducing reactive oxygen species (ROS) [17]. The components of *Cornus officinalis* showed significant free radical-scavenging activity and inhibitory effects on melanogenesis induced by radiation [11]. *Millettia dielsiana* had an anti-inflammatory effect, decreasing NO production [8]. The extracts of *Fructus crataegi* were reported to have a radio-protective effect with antioxidant activity [12,13] and protected lymphocytes from the effects of radiation [10]. *Citri reticulatae pericarpium* possessed various pharmacological effects involved in antioxidant ability against hydroxyl-induced DNA damage [9]. *Anemarrhenae* *Rhizoma* showed various bioactivities, such as anti-tumor, anti-oxidation, anti-microbial, anti-virus, anti-inflammation, anti-osteoporosis, anti-skin aging and damage effects, as well as other activities [18]. The combination of different types of medical herbs above in HSC can benefit from each other with different roles in the formula, and ultimately gain the goal of enhancing efficacy, which caters to the core thinking of traditional Chinese medicine theory.

The present study revealed that pre-administration of HSC reduced the radiation sickness characteristics and improved the 30-day survival of the irradiated rats and mice (Figure 1). In addition, the administration of HSC also increased the 30-day survival of mice after exposure to lethal TBI (Figure 1D). Our study clearly demonstrated that acute, lethal TBI of different organs was associated with significant ultrastructural changes indicative of cell damage. It also indicated that the administration of HSC was able to minimize these changes (Figure 2), confirming the protective effect of HSC on radiation-induced tissue injury. Gene expression profiles revealed a dramatic effect of HSC on alterations of gene expression caused by lethal TBI. Pretreatment with HSC prevented the differential expression of 66% (1398 genes) of 2126 genes differentially expressed in response to radiation. We focused on these genes regulated by radiation and the alterations of expression were abolished or attenuated by HSC pretreatment, as these genes might be regulated by HSC and involved in the protective effects on radiation. GO ontology analysis indicated that these genes were mainly involved in the highly enriched terms such as binding and catalytic activity for molecular function, cell parts, and cells for cellular components, cellular processes, and biological regulations for biological processes. Pathway enrichment analysis indicated that these genes were mainly involved in a total of 32 pathways (Table 1). Most of these pathways were reported to be induced by radiation in the previous studies. The pathways include olfactory transduction [19], cytokine-cytokine receptor interaction [19], neuroactive ligand-receptor interaction [20], pathways in cancer [21,22,23], MAPK signaling pathways [24], PPAR signaling pathways [25], GnRH signaling pathways [26], Notch signaling pathways [17,27,28], calcium signaling pathways [22], Wnt signaling pathways [29,30], and Jak-STAT signaling pathways [31], suggesting that HSC has radio-protective effects mainly or partially through the modulation of these pathways. For example, the Wnt pathway was activated by ionizing radiation [30]. Here, all the gene expressions involved in the Wnt signaling pathway (Table 5) were evidently up-regulated by radiation and these up-regulations were abolished or attenuated by pretreatment with HSC. Changes in Notch signaling were previously identified under the action of ionizing radiation [28]. Here, five genes were involved in the Notch signaling pathway (Table 6). Four of these gene expressions were evidently up-regulated by radiation and these up-regulations abolished or were attenuated by pretreatment with HSC. In addition, metabolomic studies demonstrated that HSC could restore the metabolic pathways perturbed by radiation, such as fatty acid metabolism, purine metabolism, tryptophan metabolism, and phenylalanine metabolism [15]. Here, our analysis indicated that the pretreatment of rats with HSC modulated radiation-induced fatty acid metabolism, purine metabolism, glutathione metabolism, starch and sucrose metabolism, and glutathione metabolism, suggesting that HSC might provide protective effects partially through the modulation of these metabolic pathways.

Since ionizing radiation induces the simultaneous compensatory activation of multiple MAPK pathways [24], we focused on these pathways. These pathways played critical roles in controlling cell survival and repopulation effects following irradiation, in a cell-type-dependent manner [24]. The 10 genes presented in Table 2 were involved in the MAPK pathways. Eight of the gene expressions were evidently up-regulated by radiation and these up-regulations were abolished or attenuated by pretreatment with HSC. These radiation-induced genes included myelocytomatosis oncogene (Myc), phospholipase A2, group IIA (Pla2g2a or sPLA2), mitogen-activated protein kinase 4 (Map4k4), protein kinase X-linked (Prkx), voltage-dependent calcium channel R type α 1E subunit (Cacna1e), voltage-dependent calcium channel L type α 1S subunit (Cacna1s), TAO kinase 2 (Taok2), and activating transcription factor 4 (Atf4). In contrast, the expressions of neurotrophic tyrosine kinase receptor type 1 (Ntrk1) and apoptosis signal-regulating kinase 1 (Ask1) were down-regulated by radiation and these down-regulations were abolished or enhanced by pretreatment with HSC. Interestingly, activating transcription factor 4 (Atf4) was a member of the ATF/CREB (activating transcription factor/cyclic AMP response element binding protein) family of basic region-leucine zipper (bZip) transcription factors. Atf4 regulated the expression of genes involved in oxidative stress, amino acid synthesis, differentiation, metastasis, and angiogenesis [32]. As described by the earlier reports [33,34], our data indicated that TBI increased the expression of Atf4. Myc was involved in a wide range of cellular processes including cell-cycle control, metabolism, signal transduction, self-renewal, maintenance of pluripotency, and control of cell fate decisions [35]. Here, our data indicated that the radiation-induced increase in Myc was attenuated by pretreatment with HSC. This was consistent with previous reports that irradiation significantly increased Myc [36,37]. Prkx was a member of an ancient family of cAMP-dependent serine/threonine kinases distinct from the classical PKA, PKB/Akt, PKC, SGK, and PKG families [38]. As described by the earlier reports [39,40], Prkx protein kinase was up-regulated by radiation. Ntrk1 was a member of the neurotrophic tyrosine kinase receptor (NTKR) family. The presence of Ntrk1 led to cell differentiation and might play a role in specifying sensory neuron subtypes [41,42]. In UV-irradiated normal skin, there was a significant reduction in Trk A/Ntrk1 tyrosine kinase receptor immunostaining after UV irradiation [43]. Though our data strongly supported the radiation-induced down-regulation of Ntrk1 [43], there were also reports on UV-induced up-regulation of both nerve growth factor NGF and its high-affinity receptor Ntrk1 [44]. cPLA2 was a member of the PLA2 enzyme super-family, which included secretory PLA2 (sPLA2), cytosolic PLA2 (cPLA2), and other members. cPLA2, which activated AA hydrolysis, existed in three isoforms: α, β, and γ. cPLA2-α was known to be a major component of the arachidonate-releasing signal transduction pathway [45,46]. Low-level laser irradiation significantly inhibited phospholipase cPLA2-α mRNA expression, which was increased in response to mechanical stress [45]. Here, our data showed that the radiation-induced increase in sPLA2 was attenuated by pretreatment with HSC. Taken together, these results indicated that the pretreatment of rats with HSC modulated multiple lethal TBI-induced MAPK pathways, suggesting that HSC might provide the protective effects partially through the modulation of MAPK pathways. These findings will help us better understand the protective effects of HSC on lethal TBI damage and provide new insights into the molecular mechanism underlying the radio-protective role of HSC in rats and create a basis for furthering our knowledge about radiation-induced molecular and cellular pathways.

## 4. Materials and Methods

### 4.1. Animals

Male ICR mice (20–22 g, 6–8 weeks old) and male Wistar rats (200–220 g) were purchased from SLAC Laboratory Animal Co., Ltd. (SLAC, Shanghai, China) and divided randomly into several groups. Animals were kept under standard laboratory conditions of temperature, pressure, and humidity. Food and water were sterilized by ^60^Co γ-irradiation and high pressure, respectively. All animal procedures were performed according to protocols approved by Institutional Animal Care and Use Committees of the Second Military Medical University (Shanghai, China). Research was conducted according to the Guide for the Care and Use of Laboratory Animals prepared by the Centre of Laboratory Animals of the Second Military Medical University. All efforts were made to minimize animal suffering.

### 4.2. Radiation and Administration

The animals were randomly assigned to one of the six following treatment groups (10–12 animals per group): normal control, HSC-High, radiation and HSC-Low, Medium, and High dose (2.5, 5, 10 g crude drug/kg body weight/day) + radiation. HSC was purchased from Shanxi Xiangju Pharmaceutical Co., Ltd. (Shanxi, China). Different doses of HSC dissolved in double-distilled water were administered intragastrically to the male animals for three consecutive days before or after irradiation. All animals, except the normal control group, were placed in specially designed, well-ventilated acrylic containers and subjected to total-body irradiation (TBI). Radiation was delivered by the ^60^Co source (Radiation facility, the Second Military Medical University, Shanghai, China). Radiation doses were 6, 6.5, 7, 8, and 8.5 Gy at a rate 2 Gy/min. The animals were monitored daily for the development of symptoms of radiation sickness and mortality.

### 4.3. Survival Assays

Wistar rats (*n* = 10 rats/group) were administered HSC before exposure to TBI with 6.0 and 7.0 Gy. ICR mice (*n* = 11–12 mice/group) were administered HSC after or before exposure to TBI with 8.0 or 8.5 Gy, respectively. We observed animals twice or three times daily for a period of 30 days to determine survival rates, and moribund animals were euthanized according to humane endpoints. The clinical criteria of moribund is being in the state of dying with no expectation of recovery, where animals display a combination of the following: lowered body temperature, continuous shaking, hunched back, impaired or slow motion, and inability to maintain sternal recumbency [47]. Moribund animals were placed in a separate cage with CO_2_ until no breathing was observed, followed by a cervical dislocation as a secondary confirmatory method of euthanasia. Any surviving animals at the end of the study were also subjected to euthanasia by the application of CO_2_ followed by cervical dislocation.

Survival of rats or mice from all the different groups was monitored from the day of onset of the experiment until the 30th day. The probabilities of the survival of all different groups were plotted as Kaplan-Meier survival curves until the 30th day. Significant differences in the survival curves among the different groups were evaluated using the log rank test (Mantel-Cox test) for multiple groups.

### 4.4. Sample Collection

Twenty-four hours following irradiation, the rats were subjected to anesthesia and then sacrificed by cervical dislocation. Thymus, spleen, liver, small intestine, testis, hippocampus, and cortex were dissected out from each animal. The specimens of the all groups were processed for ultrastructural examination. In addition, liver tissues of the all groups were processed for the microarray experiment.

### 4.5. Transmission Electron Microscopy

Transmission electron microscopy was performed essentially as described previously [48]. Biopsy samples were cut and fixed in 2.5% glutaraldehyde in 0.1 M sodium cacodylate, then post-fixed with 1% osmium tetroxide. The specimens were dehydrated through a graded series of ethanol and then embedded in labeled capsules with freshly prepared resin and left to polymerize. Ultrathin sections were cut on a Reichert Ultracut UCT (Leica, Wetzlar, Germany), stained with uranyl acetate and lead citrate and examined by a JEOL 1200EX transmission electron microscopy (Peabody, MA, USA).

### 4.6. Gene Expression Microarray and Data Analysis

The microarray experiments were performed as described previously [49]. The 4 × 44 K Whole Rat Genome Oligo Microarray (Agilent Technologies, Santa Clara, CA, USA) was hybridized with Cy3-labeled cRNA using Gene Expression Hybridization Kit (Agilent Technologies) in Hybridization Oven (Agilent Technologies), according to the manufacturer’s instructions. Raw data were obtained by Feature Extraction software 10.7 (Agilent Technologies) and normalized by Quantile algorithm, Gene Spring Software 11.0 (Agilent Technologies). The microarray experiments were conducted at the National Engineering Center for Biochip in Shanghai, China, according to the procedures in the Agilent technical manual. After normalization, genes in the treatment groups with at least 1.5-fold change in expression were considered as up-regulated or down-regulated in comparison to non-treated groups (control). To determine significant proportions of differentially expressed genes within functional groups, the hypergeometric probability *p* was calculated. *p* < 0.05 was considered significant.

Microarray data analysis was performed using the SBC Analysis system, which is available on the website: http://sas.ebioservice.com/protal/root/molnet_shbh/index.jsp. The username and password to access this website are available upon request. A general description of the SBC analysis system can be found on the website: http://www.ebioservice.com/eng/index.asp. The microarray data generated in this study have been deposited in the Gene Expression Omnibus (GEO) database under the accession number GSE57692.

### 4.7. Quantitative Real-Time (qRT)-PCR Array Validation

qRT-PCR was performed essentially as described previously [49]. In total, eight genes were chosen for RT-PCR validation. PCR primers (Table 7) were designed to either span or flank introns by using the ProbeFinder version 2.50 (http://www.roche-applied-science.com). Data are presented as mean ± SD.

**Table 7:** Oligonucleotides primers used in this study for quantitative real-time-PCR analysis. The primer sequences were designed by using the ProbeFinder version 2.49 (http://www.roche-applied-science.com).

Gene ID	Symbol	Primer	Sequence
79255	Atf4	Forward	tcagacaccggcaaggag
		Reverse	gtggccaaaagctcatctg
682930	Cacna1s	Forward	gcgtcgtggagtggaaac
		Reverse	ctctggcatgggcaggta
24577	Myc	Forward	gaatttttgtctatttggggaca
		Reverse	gcatcgtcgtgactgtcg
114850	Wnt7a	Forward	ccctcatgaacttacacaataacg
		Reverse	tggcacttacactccagtttcat
501563	Prkx	Forward	gcctgggcaacatgaaga
		Reverse	tccacacctcggaaccac
29201	Ctf1	Forward	tcctgaccaaatatgcagacc
		Reverse	agggctctccctgttgct
25682	Ppard	Forward	ggaccagagcacacccttc
		Reverse	gaggaaggggaggaattctg
59109	Ntrk1	Forward	cagcttctggctgtggcta
		Reverse	aagtgcaggctggctaggta
81822	β-Actin	Forward	cccgcgagtacaaccttct
		Reverse	cgtcatccatggcgaact

### 4.8. Statistical Analysis

Data were presented as mean ± SD. A one-way analysis of variance (ANOVA) was used to examine differences among untreated (Control) and treated (Radiation, HSC + Radiation) groups. Differences were considered significant when *p* < 0.05 or *p* < 0.01.

## 5. Conclusions

Pre-administration or administration of HSC reduced the radiation sickness characteristics while increasing the 30-day survival of the irradiated animals. In addition, HSC administration prior to radiation attenuated tissue damage induced by lethal TBI. Gene expression profiles revealed a dramatic effect of HSC on alterations of gene expression caused by lethal TBI. Pretreatment with HSC prevented the differential expression of 66% (1398 genes) of 2126 genes that are differentially expressed in response to radiation. Pathway enrichment analysis indicated that these genes were mainly involved in a total of 32 pathways, such as olfactory transduction, neuroactive ligand-receptor interaction, pathways in cancer, MAPK signaling pathways, calcium signaling pathways, cytokine-cytokine receptor interaction, PPAR signaling pathways, GnRH signaling pathways, Wnt signaling pathways, Jak-STAT signaling pathways, Notch signaling pathways, fatty acid metabolism, and purine metabolism. Our data indicated that the pretreatment of rats with HSC modulated lethal TBI-induced pathways, such as multiple MAPK pathways, suggesting that HSC might provide the protective effects mainly or partially through the modulation of these pathways. This study demonstrated the protective effects of HSC against lethal TBI and provided insights into the molecular mechanism underlying the radio-protective role in rats and created a basis for furthering our knowledge about lethal TBI-induced molecular and cellular pathways. Our data suggest that HSC has the potential to be used as an effective therapeutic or radio-protective agent to minimize irradiation damage.

## Supplementary Materials

Supplementary materials can be found at http://www.mdpi.com/1422-0067/16/08/18938/s1.
